# Oxygen saturation and lactate concentration gradient from the right atrium to the pulmonary artery in the immediate postoperative following cardiac surgery with extracorporeal circulation

**DOI:** 10.5935/0103-507X.20170042

**Published:** 2017

**Authors:** Juan Carlos Pendino, Leonardo Hess, Sergio Beltrame, Gonzalo Aldamiz-Echevarría Castillo, John Trujillo

**Affiliations:** 1 Unidad de Terapia Intensiva, Hospital Provincial del Centenario - Rosario, Argentina.; 2 Facultad de Ciencias Medicas, Universidad Nacional de Rosario - Rosario, Argentina.; 3 CIMA-Profisio, Facultad de Ciencias Medicas, Universidad Nacional de Rosario - Rosario, Argentina.; 4 Servicio de Cirugía Cardíaca, Hospital IDCSalud - Albacete, España.

**Keywords:** Oxygen/metabolism, Oxygen consumption/physiology, Lactate, Postoperative period, Thoracic surgery, Extracorporeal circulation

## Abstract

**Objective:**

This prospective study aimed to characterize the changes in blood lactate concentration and blood oxygen saturation in patients during the immediate postoperative period of cardiac surgery with extracorporeal circulation.

**Methods:**

Blood samples were collected from 35 patients in a rapid and random order from the arterial line and from the proximal and distal port of a pulmonary artery catheter.

**Results:**

The results showed no statistically significant differences between the blood oxygen saturation in the right atrium (72% ± 0.11%) and the blood oxygen saturation in the pulmonary artery (71% ± 0.08%). The blood lactate concentration in the right atrium was 1.7mmol/L ± 0.5mmol/L, and the blood lactate concentration in the pulmonary artery was 1.6mmol/L ± 0.5mmol/L (p < 0.0005).

**Conclusion:**

The difference between the blood lactate concentration in the right atrium and the blood lactate concentration in the pulmonary artery might be a consequence of the low blood lactate concentration in the blood from the coronary sinus, as it constitutes an important substrate for the myocardium during this period. The lack of differences between the blood oxygen saturation in the right atrium and the percentage of blood oxygen saturation in the pulmonary artery suggests a lower oxygen extraction by the myocardium given a lower oxygen consumption.

## Abstract (pt)

**Objetivo:**

Caracterizar as modificações na concentração sanguínea do lactato e da saturação de oxigênio em pacientes no pós-operatório imediato de cirurgia cardíaca com circulação extracorpórea.

**Métodos:**

Foram coletadas amostras de sangue de 35 pacientes, de forma rápida e aleatória, do acesso arterial e das portas proximal e distal de um cateter pulmonar.

**Resultados:**

Não foram verificadas diferenças estatisticamente significantes entre saturação de oxigênio no átrio direito (72% ± 0,11%) e na artéria pulmonar (71% ± 0,08%). A concentração sanguínea de lactato no átrio direito foi de 1,7mmol/L ± 0,5mmol/L, enquanto na artéria pulmonar esta concentração foi de 1,6mmol/L ± 0,5mmol/L (p < 0,0005).

**Conclusão:**

A diferença entre as concentrações sanguíneas de lactato no átrio direito e na artéria pulmonar pode ser consequência da baixa concentração de lactato no sangue do seio coronário, já que o lactato é um importante substrato para o miocárdio durante este período. A ausência de diferenças entre saturação sanguínea de oxigênio no átrio direito e na artéria pulmonar sugere extração de oxigênio mais baixa pelo miocárdio, em razão do menor consumo de oxigênio.

## INTRODUCTION

Several reports suggest that critically ill patients exhibit significant differences between their blood oxygen saturation (SaO_2_) values measured from blood samples drawn either from the right atrium or from the pulmonary artery. The differences span a range from 5 - 7%.^(1-3)^ It was suggested that the discordance may be explained by assuming that, in the right atrium of those patients, the blood is partially "de-saturated" by the very low oxygen content coming from the coronary sinus.^(1)^ At the same time, the blood lactate concentration [Lac] of coronary venous blood is very low because of the high rate of consumption by the myocardium in normal and even pathological situations, i.e., sepsis.^(4)^ The opposite occurs in conditions such as myocardial ischemia, where the glucose consumption is greater than the lactate consumption. Hence, in the ischemic condition, the concentration of lactate in the coronary sinus may be higher, although the metabolic substrate is highly dependent on the degree of ischemia.^(5)^

In a group of critically ill patients, [Lac] and SaO_2_ are lower in the blood of the pulmonary artery than in the blood of the right atrium.^(6)^ The samples were obtained through the proximal port (i.e., the right atrium) and distal port (i.e., the pulmonary artery) of a pulmonary artery catheter (PAC).^(7)^ Accordingly, this comparison between the blood oxygen saturation in the right atrium (S_ra_O_2_) and percentage of blood oxygen saturation in the pulmonary artery (S_pa_O_2_) along with the comparison between the blood lactate concentration in the right atrium [Lac]_ra_ and blood lactate concentration in the pulmonary artery [Lac]_pa_ has been used as a prognostic factor in critically ill patients.^(6)^

The metabolic state of the heart can be dramatically modified in conditions such as the postoperative period of cardiac surgery with extracorporeal circulation, exhibiting changes in oxygen consumption (VO_2_) and lactate metabolism. These modifications have already been highlighted by measurements of lactate, SaO_2_ and other substrates in the blood from the coronary sinus.^(8)^

The present study was designed to evaluate these blood parameters sampled simultaneously using the two ports of a pulmonary artery catheter, giving access to blood from the pulmonary artery and from the right atrium.

The aim of this study to characterize the changes in [Lac] and SaO_2_ in patients during the immediate postoperative period of cardiac surgery with extracorporeal circulation.

## METHODS

This prospective study was performed in the intensive care unit (ICU) from Hospital Idcsalud Albacete (Albacete, Spain). After approval from the Institutional Review Board of hospital (approval number 11/10), 35 patients were enrolled. The inclusion criteria were admission to the ICU shortly after cardiac surgery with extracorporeal circulation, age older than 18 years, PAC and arterial peripheral catheter insertion in the operating room. The exclusion criteria included uncorrected valve dysfunction and intracardiac communication. Informed consent was waived.

In all cases, the cardioplegia used was hematic with mild hypothermia (32°C), and the decision to use antegrade or retrograde cardioplegia depended on the type of surgery performed.

During the admission to the ICU, the medical team immediately verified the correct positioning of the PAC. This was done by checking that a wedge pressure tracing was obtained when the balloon was inflated with a volume between 0.5 and 0.8mL. Afterwards, the balloon was deflated, and the normalization of the pulmonary artery pressure tracing was verified. In addition, the right atrial pressure tracing was verified to be obtained when the proximal port of the pulmonary artery catheter was connected to the transducer. Lastly, an X-ray examination confirmed that the catheter tip was at the pulmonary hilum, at a distance no more than 2cm from the cardiac silhouette.

Immediately after checking the pressure tracing, blood samples were drawn in a rapid succession and randomly from the arterial catheter and from the proximal and distal ports of the PAC. Under these conditions, blood drawn from the proximal port was assumed to be representative of right atrium blood, whereas distal port blood was regarded as representative of pulmonary artery blood. To avoid contamination of the blood with the continuous washing solution, the first 2mL was discarded for the PAC, as well as the initial 5mL for the arterial line.

Samples for blood gas assessment were extracted in syringes specific for this purpose (Pulsettm Westmed, Tucson, AZ, USA). Blood samples for assessing [Lac] were extracted using ad hoc tubes with sodium fluoride and potassium oxalate. The blood samples were processed immediately. The oxygen saturation was measured using the Nova Biomedical Phox Plus^®^ analyzer (Waltham, MA, USA). Lactate determination was performed with a standard clinical laboratory instrument (Dade Behring Dimension^®^ RxL analyzer) (Deerfield, IL, USA). The routine laboratory tests for the immediate postoperative control include hematocrit, white blood count, coagulation tests, blood glucose, plasma urea, plasma creatinine, serum sodium, serum potassium, serum chloride, serum magnesium, serum phosphorus, plasma creatine kinase (CK), plasma CK-MB and plasma troponin.

The cardiac output was measured by the thermodilution method as the average of five sequential determinations. The right atrium pressure, the pulmonary artery pressure and the pulmonary artery occlusion pressure (PAOP) were also measured employing standard methods. Oxygen transport (DO_2_), VO_2_, oxygen extraction ratio (O_2_ER), double product and index of left ventricular stroke work (LVSWI) were calculated using conventional formulae. Finally, the central temperature was obtained through the thermistor of PAC, shortly after connection to the monitoring system. The preoperative left ventricular ejection fraction was assessed in 32 of 35 patients, either by two-dimensional echocardiography or cardiac catheterization.

### Statistical comparisons

Paired Student's *t*-test was used to compare atrial *versus* pulmonary artery measurements. Spearman correlation analysis was performed to compare [Lac]_ra_ and [Lac]_pa_. The method of Bland & Altman was used to investigate the effect of [Lac] on the differences between pairs of observations. The relation between Δ[Lac] and ΔSaO_2_ and other hemodynamic parameters (cardiac output, double product, LVSWI, DO_2_, VO_2_ and O_2_ER) was analyzed by employing the Spearman correlation test. Data are shown as the mean ± standard deviation (SD). The level of statistical significance was set at p < 0.05.

## RESULTS

A group of 35 patients, 19 males and 16 females, aged 67.7 ± 10 years, was enrolled in the study. The preoperative left ventricular ejection fraction was 52.81% ± 13.1%. Ten of 35 patients underwent coronary artery bypass graft (CABG), 8 patients received valve prostheses at the mitral position (MVR), 10 patients received valve prostheses at the aortic position (AVR), 4 patients underwent concomitant MVR + AVR, 2 patients were subjected to simultaneous CABG + MVR, and the remaining patient received a Bentall prosthesis. The time elapsing between the aortic unclamping blood sample collection and hemodynamic measurements in the ICU was 59.4 minutes ± 11.2 minutes. Hemodynamic parameters, Hb values, cardiopulmonary bypass time, central temperature and demographics data are shown in table 1.

**Table 1:** Demographic and hemodynamic parameters

Patient parameters (N = 35)	Mean ± SD
Sex (male/female)	19/25
Age (years)	67.8 ± 1.70
APACHE II Score	13.3 ± 1.35
Ejection fraction (%)	52.81 ± 13.10
Heart rate (beats/minute)	82.2 ± 2.84
MAP (mmHg)	73.3 ± 2.67
MPAP (mmHg)	24.4 ± 1.32
PAOP (mmHg)	15.1 ± 0.91
RAP (mmHg)	10.9 ± 0.71
Cardiac output (L/minute)	4.73 ± 0.26
Cardiac index (L/minute/m^2^)	2.74 ± 0.14
DO_2_ (mL/min)	693 ± 42.1
VO_2_ (mL/min)	211 ± 12.5
O_2_ER (%)	31.7 ± 1.36
LVSWI (g/m^2^/beat)	37.0 ± 3.32
Double product (systolic blood pressure × heart rate)	9989 ± 380
SVRI (dynes/seconds/cm^-5^)	2245 ± 125
Hemoglobin concentration (grams%)	10.5 ± 0.24
Central temperature (degrees Celsius)	35.3 ± 0.13
ECT (minutes)	84.6 ± 5.19
ACCT (minutes)	57.2 ± 4.87

SD - standard deviation; APACHE II - Acute Physiology and Chronic Health Evaluation II; MAP - mean arterial pressure; MPAP - mean pulmonary artery pressure; PAOP - pulmonary artery occlusion pressure; RAP - right atrial pressure; DO_2_ - oxygen delivery; VO_2_ - oxygen consumption; O_2_ER - oxygen extraction index; LVSWI - left ventricular stroke work index; SVRI - systemic vascular resistance index; ECT - extracorporeal circulation time; ACCT - aortic cross-clamp time.

**Figure 1:**
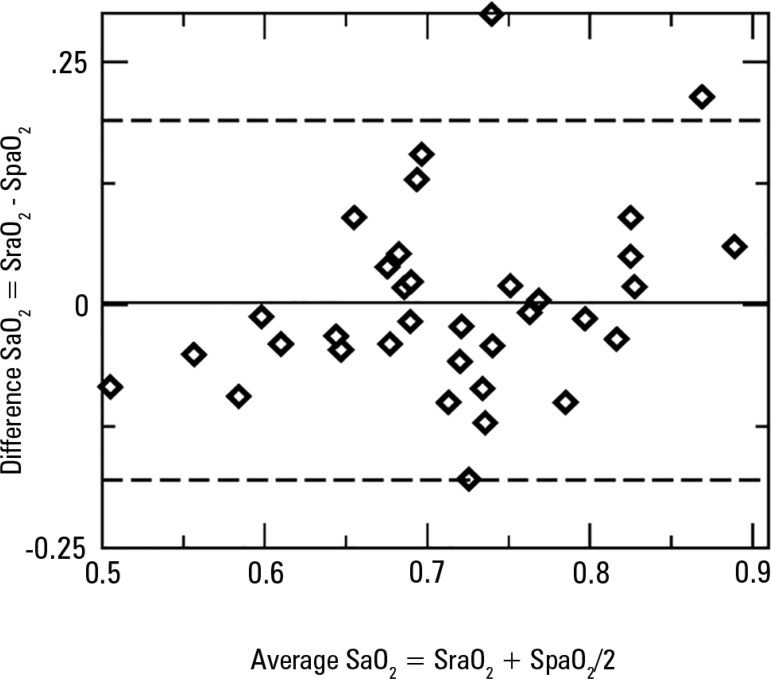
Bland & Altman plot comparing oxygen saturation in the right atrium and oxygen saturation pulmonary artery. SaO_2_ - blood oxygen saturation; SraO_2_ - oxygen saturation in the right atrium; SpaO_2_ - oxygen saturation pulmonary artery.

**Figure 2:**
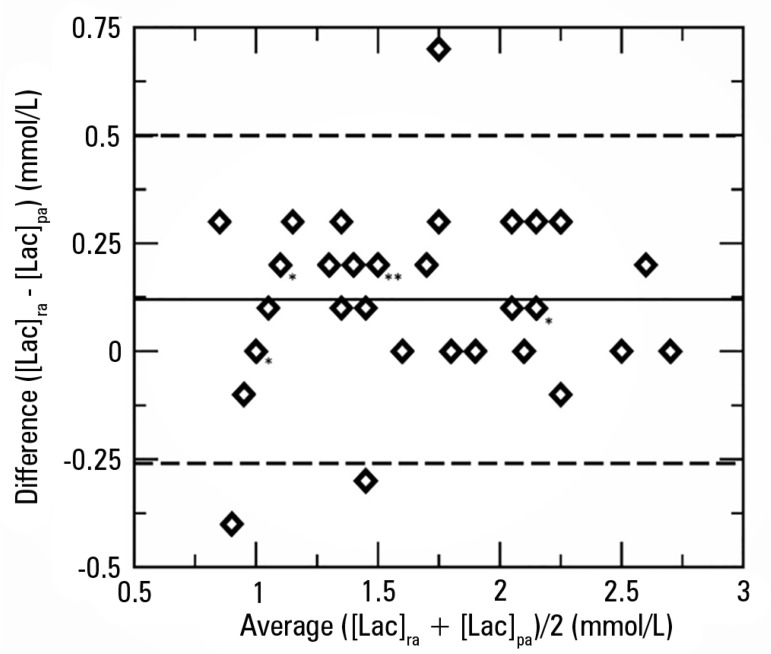
Bland & Altman plot comparing lactate concentration in right atrium and lactate concentration in pulmonary artery. [Lac]_ra_ - lactate concentration in right atrium; [Lac]_pa_ - lactate concentration in pulmonary artery. * Superposition of patients with similar values corresponding to lactic acid measurements.

There were no statistically significant differences between the S_ra_O_2_ and S_pa_O_2_. The [Lac]_ra_ was higher than [Lac]_pa_ (p < 0.0005) with a gradient of 0.1mmol/L ± 0.2mmol/L (Table 2). There was no significant correlation between Δ[Lac] and any of the following parameters: cardiac output, double product, LVSWI, DO_2_, VO_2_ and O_2_ER. The Bland & Altman test for SO_2_ and [Lac] showed a bias of 0.00061 (95%CI -0.185169 to 0.186391) and 0.1 for [Lac]_ra_ (95%CI -0.25092 to 0.50092), respectively (Figures 1 and 2). Analysis on the relationship between the preoperative ejection fraction and age, extracorporeal circulation time, ICU stay, DO_2_, VO_2_ and IEO_2_, remained insignificant.

**Table 2:** Oxygen saturation and lactate concentration of paired right atrium and pulmonary artery blood samples

	Right atrium blood	Pulmonary artery blood	Gradient (Δ right atrium - pulmonary artery)
SaO_2_ (%)	71.15 ± 1.88	71.09 ± 1.43	0.103 ± 1.59
[Lac] mmol/L	1.772 ± 0.1148	1.647 ± 0.1114*	0.125 ± 0.032

SaO_2_ - blood oxygen saturation; [Lac] - blood lactate concentration;

* p < 0.001 when comparing atrial to mixed venous blood by paired t-test. Averages ± standard deviation.

## DISCUSSION

Our first working hypothesis was that there should not be a SaO_2_ gradient between the blood of the right atrium and pulmonary artery because there is expected to be a low oxygen extraction by the myocardium during the immediate postoperative period. Alternatively, there should be a gradient of lactate between the blood from the right atrium and the pulmonary artery because the myocardium, under these conditions, would be able to use additional lactate as the preferential substrate.

Two main findings are in line with our initial hypothesis. The first one is the presence of a gradient in the concentration of lactate in paired samples obtained from the proximal and distal ports. The second one addresses the lack of differences between S_ra_O_2_ and S_pa_O_2_.

The drop in [Lac] between blood from the right atrium and blood from the pulmonary artery may be due to the blood supply with low concentration of lactic acid from the coronary sinus. Free fatty acids and lactate are the primary substrates used by the heart in normal conditions. During aortic clamping and despite myocardial protection with electromechanical cardiac arrest and hypothermia, the heart can suffer from ischemia. During the first hour following the placement of the aortic clamp in CABG, the myocardium oxidizes more glucose and less free fatty acids than in preoperative period and 6 hours after CABG surgery. When the aortic cross clamp is removed, the myocardium might extract more high energy substrates, presumably reflecting its accumulation during extracorporeal circulation.^(8)^ This immediate period following separation from extracorporeal circulation is accompanied by a hyperemic response evidenced by a progressive increase in coronary blood flow and, furthermore, a decrease in the oxygen extraction ratio due to a limited ability to use oxygen.^(8,9)^ In addition, the lactate extraction ratio increases progressively from the time of release of the aortic cross-clamp.^(8)^

It seems sensible to expect a decrease in the myocardial oxidative activity as the protection exerted by hypothermic cardioplegia. This situation then allows for the accumulation of lactate in the context of myocardial oxygen deprivation. Because lactate can be easily metabolized to pyruvate; the former may be then the preferred substrate for aerobic metabolism after recovery post cardiac arrest, when surgery is accomplished. However, we are not able to explain how the myocardium may use lactate without a corresponding increase in oxygen utilization. Sobrosa et al.^(10)^ found positive gradients between arterial blood lactate and blood from the coronary sinus, concomitantly with a minimum difference between the SO_2_ of arterial blood and the blood from the coronary sinus in patients undergoing CABG with cardiopulmonary bypass at the time of reperfusion.

These changes may partly be explained by the transient depression of the myocardial capacity of extraction, described during early reperfusion.^(11)^

The variables analyzed by our group are the macrocirculatory ones assessing the overall metabolic status of tissues such as the myocardium, but it is unknown what occurs at the cellular level.

The presence of a cytoplasmic-to-mitochondrial lactate shuttle in the heart allows glycolysis to progress to lactate without the adverse consequence of acidosis or altered redox.^(12)^ In isolated rat hearts with ischemia-reperfusion injuries, monocarboxylate transporters (MCT) isoform 4 was significantly increased following global ischemia, as was MCT isoform 1 expression during the early stages of reperfusion. Increased MCT4 expression may facilitate lactate extrusion during the ischemic period, while increased MCT1 may favor lactate transport into and out of cells simultaneously during early reperfusion.^(13)^

Rao et al. observed^(14)^ that the persistence in the release of lactate by the coronary sinus during reperfusion suggests a late recovery in myocardial aerobic metabolism likely related to inadequate protection during cardiopulmonary bypass as well as to impaired functional contractility of the heart and low cardiac output syndrome.

Caution should be exerted when comparing to the study by Gutierrez et al.,^(7)^ in which blood samples were obtained at different times, when it was decided that a PAC was necessary to guide fluid therapy. Our study appears more homogeneous in terms of the underlying process and the time point at which the hemodynamic and laboratory tests were performed.

Right atrial blood is a mixture of superior vena cava and inferior vena cava blood. It is possible that the blood from the superior vena cava and inferior vena cava had not fully mixed at the proximal port of the PAC, and if so, the mixture of blood may have occurred distally to the proximal PAC sampling port. An answer to this question can only be achieved by direct measurement of [Lac] from the inferior vena cava inferior vena cava to the pulmonary artery.

Only four patients from our series had [Lac]_pa_ higher than [Lac]_ra_. This may be due to technical errors or catheter position, or because they experienced myocardial ischemia. In this regard, the [Lac] in the coronary sinus was high because this condition was associated with the release of lactate by the coronary sinus serving to explain differences. In these 4 patients who underwent heart valve replacement, there was no evidence of myocardial ischemia, in light of the usual methods for detecting ischemia at the bedside. Notably, 3 of them required a temporary pacemaker because they exhibited conduction disturbances during the immediate postoperative period. One of these four patients required norepinephrine at the end of surgery to maintain an adequate MAP, being the only one in need of vasoactive drugs among the total population.

Notably, the device employed for lactate measurements has a precision of ± 0.09mmol/L. Hence, when assuming a systematic instrumental bias, the difference in [Lac] between the right atrium and the pulmonary artery would have remained statistically significant.

We found no differences in the SaO_2_ between the right atrium and pulmonary artery, although the Bland-Altman test indicated that both variables were not interchangeable. The wide 95% limits of agreement between central venous and pulmonary artery SaO_2_ might be accounted for by dissimilar individual behaviors.

As above stated, subsequent to the release of the aortic cross-clamp, there may be a decrease in oxygen extraction by the myocardium, most likely related to mitochondrial dysfunction.

The oxygen heart demand is closely related to the myocardial work.^(4)^ The double product (an indirect measure of VO_2_ by the myocardium) and LVSWI were within normal ranges in our patient series.

Moreover, the total body metabolic demand is decreased during the immediate postoperative period of patients undergoing hypothermic cardiopulmonary bypass,^(15)^ reflected in a reduction in systemic VO_2_ and carbon dioxide production. These findings are related to deep sedation, mechanical ventilation and mild hypothermia. Hence, the lack of difference between SraO_2_ and SpaO_2_ may be related to lower myocardial oxygen consumption in these patients.

## CONCLUSION

Differences were found in the blood lactate concentration between the right atrium and the pulmonary artery. This may be due to a low-lactate blood supply from the coronary sinus, in turn suggesting that the myocardium may preferentially use lactate as a substrate in that situation. The lack of difference between the blood oxygen saturation in the blood of the right atrium and pulmonary artery may be explained by a lower myocardial oxygen extraction.
